# Phonon mechanism of compressive strain enhanced thermal transport in defective high entropy alloy heterojunctions

**DOI:** 10.1186/s11671-026-04823-z

**Published:** 2026-08-20

**Authors:** Mingjian Zhou, Yunqing Tang, Xingang Ren, Ao Yang, Zheng Zhang

**Affiliations:** 1 School of Mechanical Engineering, Chaohu University, Chaohu, 238000 China; 2 School of Mechanical Engineering, Shandong University, Jinan, 250061 China; 3 School of Electronic and Information Engineering, Anhui University, Hefei, 230000 China

**Keywords:** High-entropy alloy/graphene, Compressive strain, Molecular dynamics, Interfacial thermal regulation, Phonon

## Abstract

High-entropy alloy (HEA)/graphene (Gr) heterojunctions hold significant promise for thermal management in micro/nanoelectronic devices, yet the interfacial defect and thermal transport regulation mechanisms remain to be further elucidated. Using non-equilibrium molecular dynamics simulations, this study systematically investigates the through-plane thermal conductivity and interfacial thermal conductance (ITC) of HEA/Gr/HEA heterojunctions containing nanoscale void defects, and explores the regulatory effect of compressive strain on interfacial thermal transport and its microscopic phonon mechanisms. The results show that void defects significantly suppress thermal transport: the ITC is approximately 212.3 MW/m^2^K in the defect-free case and decreases to about 139.6 MW/m^2^K (a reduction of 34.2%) when the number of nanopores increases to nine. Within the elastic strain range of 0–4%, compressive strain effectively enhances ITC; as the strain increases from 0 to 4%, ITC rises from 185.2 MW/m^2^K to 353.4 MW/m^2^K (an increase of 90.8%). Applying only 2% strain completely compensates for the thermal conductivity degradation induced by void defects. Phonon density of states analysis reveals that compressive strain increases the PDOS of the Gr layer in the low-frequency region (0–15 THz) while decreasing that of the HEA layer, thereby improving their phonon spectral overlap. The phonon energy coupling coefficient increases from 0.44 to 0.53 (a 20.45% enhancement), mainly originating from strengthened coupling in the 5–20 THz frequency range. This study elucidates the phonon physics mechanism underlying the enhancement of interfacial thermal transport performance, providing a theoretical foundation for thermal management of HEA/Gr heterojunctions and strain-modulated interfacial thermal resistance.

## Introduction

With the continuous increase in integration density of micro/nanoelectronic devices, interfacial thermal resistance has become one of the critical bottlenecks limiting device performance and reliability [1–4]. High-entropy alloys (HEAs) exhibit broad application prospects in micro/nanoelectronic packaging and thermal management owing to their excellent mechanical properties, thermal stability, and tunable physicochemical characteristics [5–12]. Graphene (Gr), as a two-dimensional material with exceptionally high intrinsic thermal conductivity, is widely regarded as an ideal heat-dissipating material [13–17]. Therefore, constructing HEA/Gr heterojunctions, which combine the structural stability of HEA with the efficient thermal transport capability of Gr, is expected to become an important candidate system for a new generation of functional thermal management interface materials.

However, during actual fabrication and service, interfaces of HEA/Gr heterojunctions inevitably contain defects such as nanoscale voids and vacancies. These defects significantly enhance phonon scattering and reduce interfacial thermal transport efficiency [6, 18–21]. Previous studies have shown that the increase in interfacial thermal resistance induced by defects can cause local overheating of devices, severely compromising overall performance and lifetime [22]. Therefore, elucidating the suppression mechanism of defects on interfacial thermal transport in HEA/Gr heterojunctions and exploring effective thermal regulation strategies are of great scientific significance and engineering value. In recent years, mechanical strain has received extensive attention as a means of actively tuning the physical properties of materials [23]. Theoretical and experimental studies have demonstrated that tensile or compressive strain can alter atomic spacing and modulate phonon dispersion relations, thereby affecting the thermal conductivity of materials [20, 24, 25]. Particularly in van der Waals heterojunctions, applying compressive strain in the out-of-plane direction can enhance interlayer coupling and promote interfacial phonon matching, thus potentially improving interfacial thermal transport efficiency [26, 27]. However, systematic studies on the thermal transport behavior and underlying microscopic mechanisms of defective HEA/Gr heterojunctions under compressive strain are currently lacking.

Against this background, this paper employs non-equilibrium molecular dynamics (NEMD) simulations to systematically investigate the through-plane thermal conductivity and interfacial thermal conductance (ITC) of HEA/Gr/HEA heterojunctions containing nanoscale void defects, and explores the regulatory effect of compressive strain on interfacial thermal transport. The effects of void defect type and number on thermal transport performance are analyzed in detail, and the phonon mechanisms underlying the enhancement of ITC by compressive strain within the elastic range are revealed, including variations in phonon density of states overlap, phonon energy coupling coefficient, and interfacial temperature jump. The findings aim to provide theoretical guidance for the application of HEA/Gr heterojunctions in thermal management of micro/nanoelectronic devices and to offer new insights into tuning interfacial thermal resistance via strain engineering.

## Models and methods

### Model construction and simulation methods

**Fig. 1:**
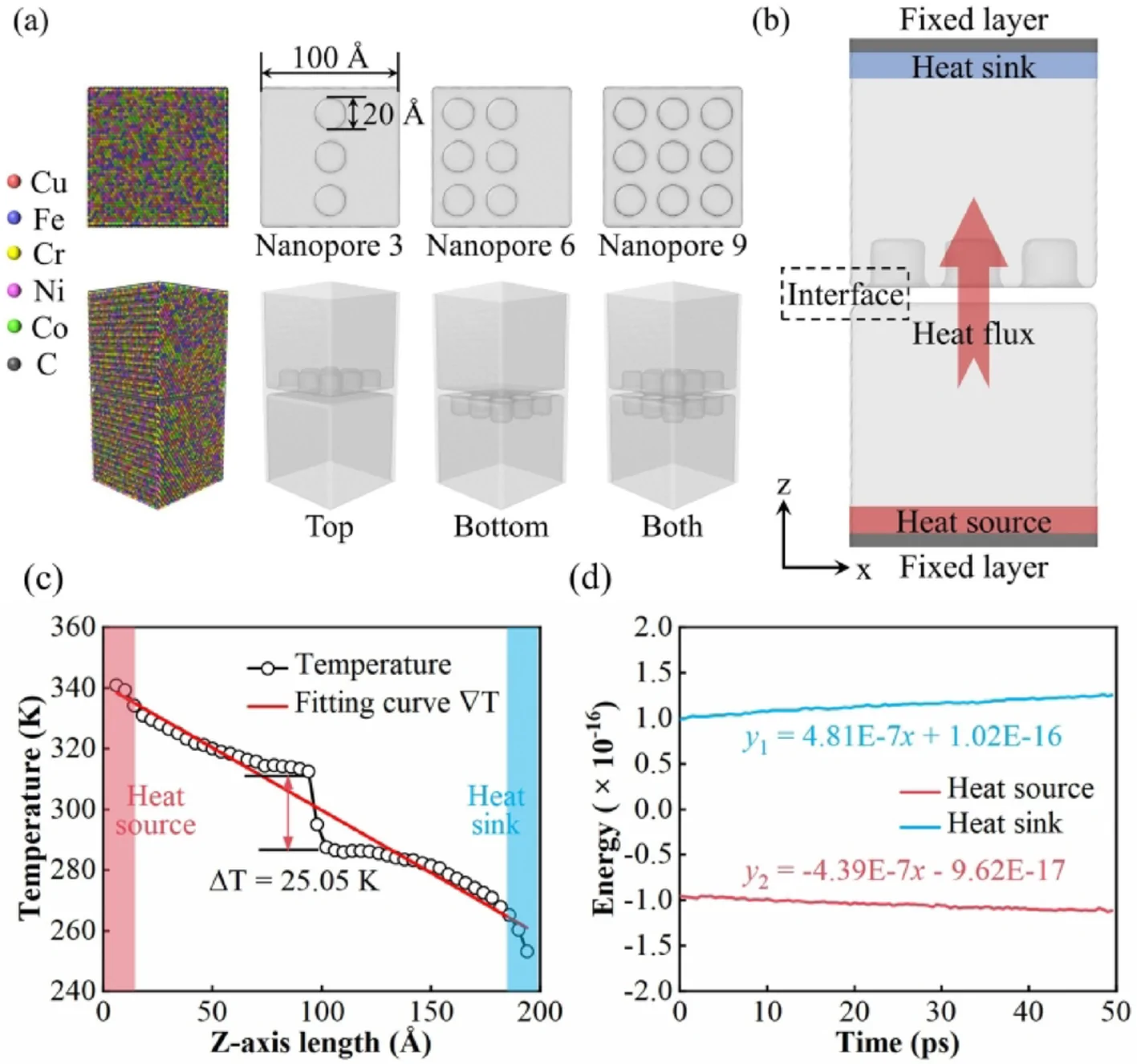
Calculation of through-plane thermal conductivity and ITC of HEA/Gr/HEA heterojunctions using the NEMD method.** a** schematic structures of perfect and nanopore-defective HEA/Gr/HEA heterojunctions.** b** a schematic representation of the NEMD simulation workflow. The system temperature gradient under steady state is typically depicted in (**c**). Furthermore, the cumulative exchange energy as a function of time is plotted in (**d**), from which the energy exchange rate *E*(*t*) is determined. Specifically, by taking the mean of the absolute slopes of the two curves shown in (**d**)

The Large-scale Atomic/Molecular Massively Parallel Simulator (LAMMPS) was used to perform all atomic modeling and molecular dynamics simulations. The simulation system measures 100 Å × 100 Å × 200 Å in the X, Y, and Z directions. We further verified that varying the simulation box length (150 Å, 200 Å, 250 Å) along the heat flux direction changes the ITC by less than 5%, illustrated in Table 1, confirming that size effects are negligible and the 200 Å system captures intrinsic thermal transport [7, 28]. The high-entropy alloy is an equiatomic FeNiCrCoCu single-phase alloy with a face-centered cubic (FCC) lattice. It has been shown that a 200 Å length along the heat flux direction (Z-axis) is adequate for qualitative thermal transport studies [7, 25, 29]. The constructed heterostructure consists of alternating HEA and graphene layers separated by 3.4 Å, and the schematic is given in Fig. 1a. When void defects are present, they are modeled as cylindrical nanopores (diameter = 20 Å, height = 20 Å) uniformly distributed on both sides of the interface. These nanopores are categorized into three types: Top, Bottom, and Both.

**Table 1:** Sensitivity of through-plane thermal conductivity (*K*_*through−plane*_) and interfacial thermal conductance (ITC)

System length (Å)	K_through−plane_ (W/mK)	ITC (MW/m^2^K)	Relative change in K_through−plane_	Relative change in ITC
150	1.19	214.8	4.8%	1.2%
200	1.25	212.3	This study	This study
250	1.22	211.4	2.4%	0.4%

The simulation configuration is HEA/Gr/HEA_pure (*ε* = 0%)

The optimized AIREBO potential parameters capture the interactions between carbon atoms in the graphene layer [14]. While the optimized Tersoff potential [30] offers higher accuracy for phonon transport in pristine graphene, we choose AIREBO because it has been extensively validated for metal‑graphene interfaces, reliably captures relative trends under defects and strain, and yields reasonable phonon density of states for comparative analysis [20, 31–34]. Meanwhile, atomic interactions within the HEA are modeled using the embedded atom method (EAM) potential [5, 35, 36], which has been benchmarked against the solid-phase mixing enthalpy and lattice constants and successfully applied to thermophysical property studies of HEAs [37, 38]. For the cross-interface coupling between graphene carbon and HEA metal atoms, the Lennard-Jones (L-J) potential is employed, a classic and computationally efficient model well suited for describing van der Waals-dominated physisorption interfaces. This potential has been widely used in molecular dynamics simulations of interfacial heat transfer in metal‑carbon nanocomposites [39, 40]. Its cutoff distance is set to 10 Å, approximately three times *σ*_C‑Cu_ = 3.2723 Å, with the relevant parameters provided in Table 2 [41]. The energy (*ε*) and distance (*σ*) parameters are derived from the Universal Force Field (UFF) together with the Lorentz-Berthelot mixing rules [42].

We acknowledge that the L-J potential provides only a qualitative description of van der Waals interactions and may not fully capture strain‑dependent changes in interfacial coupling. Nevertheless, this potential has been extensively employed in previous molecular dynamics studies of graphene‑reinforced high‑entropy alloy composites and related sandwich heterostructures to describe interfacial mechanical behavior, including dislocation nucleation, interface shear resistance, and load transfer [43–45]. Therefore, while its limitations are recognized, the present L‑J parameterization is consistent with the current literature and provides a valid basis for our comparative study. In future work, more accurate descriptions using machine‑learned interatomic potentials (MLIPs) should be pursued to capture strain‑dependent van der Waals coupling with higher fidelity.

**Table 2:** The L-J parameter between the graphene layer and the high-entropy alloy layer

Atoms	*\varepsilon * (eV)	*σ *(Å)
C-Fe	0.0016021	3.0126
C-Cr	0.0017210	3.0620
C-Ni	0.0017210	2.9778
C-Co	0.0016626	2.9948
C-Cu	0.0009936	3.2723

The simulation workflow of the NEMD method applied in this study is illustrated in Fig. 1b. This method is utilized to compute both the through-plane thermal conductivity of the HEA and the ITC [46]. Initially, periodic boundary conditions are imposed along all three spatial directions, together with a time step of 0.5 fs and an initial temperature of 300 K. To release the internal stresses accumulated during model construction, an energy minimization and system relaxation are first carried out under the isothermal‑isobaric (NPT) ensemble for 0.5 ns using the conjugate gradient algorithm. Following this, the system is switched to the canonical (NVT) ensemble for an additional 0.5 ns of relaxation, allowing it to reach thermodynamic equilibrium. Afterwards, the boundary condition along the heat flux direction (Z‑axis) is converted to non‑periodic with a shrink‑wrapped treatment. A Langevin local thermostat then maintains the two ends of the model at 340 K and 260 K, respectively, thereby creating a steady temperature gradient under the microcanonical (NVE) ensemble. A 50 ps data collection phase then follows, during which the cumulative energy exchanged between the heat bath and the heat sink is recorded. Throughout this phase, thermodynamic quantities (including temperature profiles, cumulative exchanged energy, and atomic velocities) were output every 0.5 ps (i.e., every 1000 time steps), providing sufficient temporal resolution to resolve phonon dynamics and ensure reliable steady‑state averaging [14, 47]. The sampled data are subsequently averaged statistically for the determination of thermophysical properties; typical results from this acquisition are displayed in Figs. 1c and d. Finally, the through-plane thermal conductivity is derived via the following expression:

$${K_{through-plane}}=\frac{E}{{At\nabla {\mathrm{T}}}}$$

In the equation, *K* is the through-plane thermal conductivity, *A* is the cross-sectional area perpendicular to the heat flux direction, *t* is the simulation time, *E* is the energy change of the system, and $\nabla {\mathrm{T}}$ is the temperature gradient.

ITC can be characterized by the temperature jump ΔT and the heat flux *J*(*t*), and is calculated using the following formula:

$$ \left\{ {\begin{array}{*{20}l} {{\text{ITC = }}\frac{{J(t)}}{{\Delta {\mathrm{T}}}}} \hfill \\ {J(t) = \frac{Q}{A},{\text{ }}Q = \frac{{\Delta \sum\limits_{{i \in N}} {E_{i} } }}{{\Delta t}}} \hfill \\ \end{array} } \right. $$

In the equation, *J*(t) is the heat flux, ΔT is the temperature jump, *Q* is the energy exchange rate, *E*_*i*_ is the energy of particle *i*, *N* is the number of particles in the constant-temperature region, and *t* is the simulation time.

### Computational methods for phonon properties

The phonon density of states (PDOS) itself is a key phonon property reflecting the thermal transport behavior of a material. It is obtained by applying a Fourier transform to the atomic velocity autocorrelation function:

$${\mathrm{PDOS}}\left( \omega \right)=\int_{{ - \infty }}^{{+\infty }} {\left\langle {\frac{1}{N}\sum\limits_{{i=1}}^{N} {\left[ {{{{\mathbf{\overset{\lower0.5em\hbox{$\smash{\scriptscriptstyle\rightharpoonup}$}} {v} }}}_i}\left( {t0} \right) - {{{\mathbf{\overset{\lower0.5em\hbox{$\smash{\scriptscriptstyle\rightharpoonup}$}} {v} }}}_i}\left( {t0+t} \right)} \right]} } \right\rangle } {e^{ - 2\pi i\omega t}}dt$$

In the above expression, $${{\mathbf{\overset{\lower0.5em\hbox{$\smash{\scriptscriptstyle\rightharpoonup}$}} {v} }}_i}\left( t \right)$$ denotes the velocity of the *i*-th atom at time *t*, *N* represents the total number of atoms, and *ω* stands for the phonon frequency.

In van der Waals heterostructures, the variation mechanism of ITC is often explained using the phonon energy coupling (PEC), which characterizes the vibrational modes of phonons at the heterointerface. PEC is derived from the PDOS and is calculated via the following formula:

$${\mathrm{PEC=}}\frac{{\int_{{\mathrm{0}}}^{\infty } {\hbox{min} \left\{ {{\mathrm{PDO}}{{\mathrm{S}}_{{\mathrm{Gr}}}}\left( \omega \right),{\mathrm{PDO}}{{\mathrm{S}}_{{\mathrm{HEA}}}}\left( \omega \right)} \right\}d\omega } }}{{\int_{{\mathrm{0}}}^{\infty } {\hbox{max} \left\{ {{\mathrm{PDO}}{{\mathrm{S}}_{{\mathrm{Gr}}}}\left( \omega \right),{\mathrm{PDO}}{{\mathrm{S}}_{{\mathrm{HEA}}}}\left( \omega \right)} \right\}d\omega } }}$$

The PEC is defined using the PDOS of Gr and HEA at a given frequency *ω*, denoted as PDOS_Gr_(*ω*) and PDOS_HEA_(*ω*), respectively. This approach ensures that all vibrational modes inherent to both materials are comprehensively accounted for. When the PEC value approaches 1, it indicates that the two phonon density of states curves almost completely overlap, meaning that the two materials are highly matched in the frequency and intensity of their vibrational modes. When the PEC value approaches 0, it reflects extremely low overlap of the phonon density of states curves, indicating fundamentally different vibrational characteristics between the two materials.

Phonon coupling spectral decomposition (PCSD) serves as a metric to evaluate how well the vibrational modes of two materials match each other across various frequencies within a heterostructure. Only frequencies below a certain threshold *ω* are considered. The PCSD value, denoted as *G*(*ω*), is computed using the following equation [37]:

$$G(\omega ){\mathrm{=}}\frac{{\int_{{\mathrm{0}}}^{{\bar {\omega }}} {{\mathrm{PDO}}{{\mathrm{S}}_A}(\omega ) \times {\mathrm{PDO}}{{\mathrm{S}}_B}(\omega )d\omega } }}{{\int_{{\mathrm{0}}}^{\infty } {{\mathrm{PDO}}{{\mathrm{S}}_A}(\omega )d\omega } \times \int_{{\mathrm{0}}}^{\infty } {{\mathrm{PDO}}{{\mathrm{S}}_B}(\omega )d\omega } }}$$

Here, *A* and *B* label the two components that constitute the interface. PDOS of *A* and *B* at a given frequency *ω*, denoted as PDOS_*A*_(*ω*) and PDOS_*B*_(*ω*), respectively. It is worth noting, PDOS is a scalar quantity. *G*(*ω*) specifically accounts for vibrational modes whose frequencies lie below the cutoff *ω*. The lower the value of *G*(*ω*), the greater the vibrational mismatch between the two materials.

In the frequency domain, the spectral heat flux (SHC) across the interface can be resolved by.

$$J(\omega ) \approx - \frac{2}{{{t_{{\mathrm{simu}}}}\omega }}\operatorname{Re} \sum\limits_{{i \in {\mathrm{Gr}}}} {\sum\limits_{{j \in {\mathrm{HEA}}}} {\sum\limits_{{\alpha ,\beta \in \left\{ {x,y,z} \right\}}} {\operatorname{Im} \left\langle {\hat {v}_{i}^{\alpha }{{(\omega )}^ * }K_{{ij}}^{{\alpha \beta }}\hat {v}_{j}^{\beta }(\omega )} \right\rangle } } } $$

where $\hat {v}_{i}^{\alpha }(\omega )$ and $\hat {v}_{j}^{\beta }(\omega )$ are the Fourier transform atomic velocities of atom *i* in the Gr layer and atom *j* in the HEA layer, respectively. $K_{{ij}}^{{\alpha \beta }}$ is the interface force constant matrix determined by the finite difference method, and ${t_{{\mathrm{simu}}}}$ is the correlation time between force and velocity. Based on Fourier’s law, the spectral ITC can be expressed as $T\left( \omega \right)$ by.

$$T\left( \omega \right)=\frac{{\left| {J\left( \omega \right)} \right|}}{{A\Delta {\mathrm{T}}}}$$

where *A* is the overlapping area between Gr and HEA. Here, the $\Delta {\mathrm{T}}$ is the interface temperature difference.

## Results and discussion

### Calculated through-plane thermal conductivity and interfacial thermal conductance

**Fig. 2:**
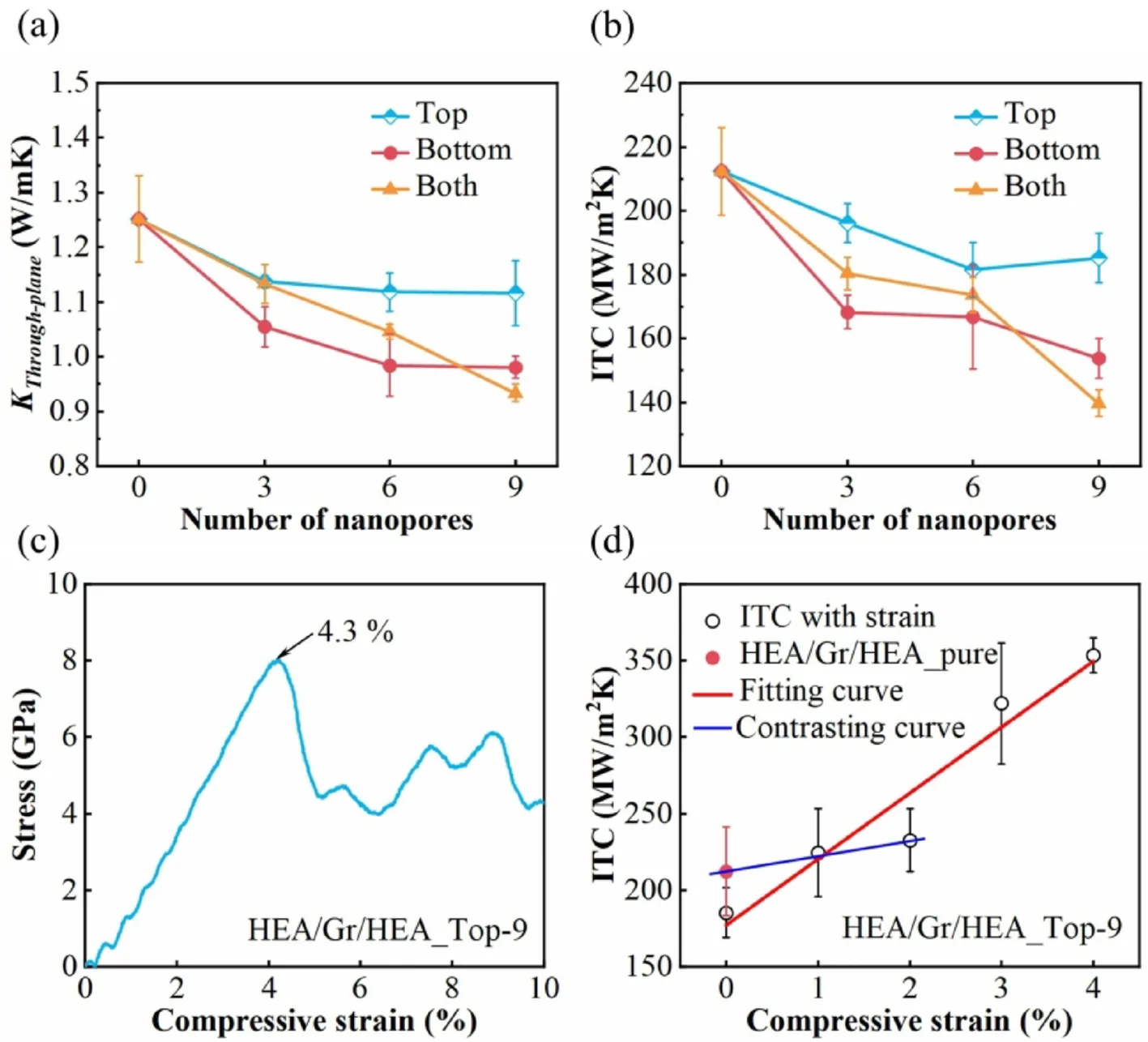
Numerical calculation results.** a** through-plane thermal conductivity and** b** ITC of HEA/Gr/HEA heterojunctions as functions of the number of nanopores for different void configurations.** c** compressive strain-stress curve of the HEA/Gr/HEA_Top-9 configuration with void defects.** d** effect of compressive strain on the ITC of the HEA/Gr/HEA_Top-9 heterojunction

The numerical results are shown in Fig. 2. The through-plane thermal conductivity results in Fig. 2a indicate that for the HEA/Gr/HEA heterojunction without nanopores, the through-plane thermal conductivity is approximately 1.25 W/mK. As the number of nanopores increases, the through-plane thermal conductivity exhibits a decreasing trend for all three void defect types: Top, Bottom, and Both. Among these, the Both void defect type shows the largest reduction in through-plane thermal conductivity. For example, when the number of nanopores increases from 0 to 9, the through-plane thermal conductivity decreases from 1.25 W/mK to approximately 0.92 W/mK, a reduction of 35.9%. The ITC data in Fig. 2b show that in the absence of nanopores (0 nanopore), the ITC on the Top, Bottom, and Both sides is approximately 212.3 MW/m^2^K. When the number of nanopores increases to 3, the ITC decreases to approximately 168.2–196.2 MW/m^2^K. When the number increases to 9, the ITC further decreases to approximately 139.6 MW/m^2^K. The analysis indicates that nanoscale void defects have a significant suppressive effect on both interfacial and through-plane thermal transport. The underlying mechanism can be explained from the perspective of phonon scattering: as geometric defects at the interface, nanopores disrupt the continuity of phonon propagation and act as strong phonon scattering centers. When phonons traverse the interface, they must detour around the void regions, resulting in longer effective heat transport paths and increased scattering probability. Furthermore, stress concentration around the nanopores induces local lattice distortion, which further enhances anharmonic phonon scattering and reduces thermal conductivity. It is noteworthy that when nanopores are present on both sides of the interface (Both configurations), the thermal transport performance decreases most significantly, indicating that the synergistic effect of defects on both sides further exacerbates thermal resistance. According to the results in Figs. 2a and b, the Top configuration with void defects exhibits higher through-plane thermal conductivity and ITC. Moreover, at the micro/nanoscale, ITC dominates the overall thermal performance. Therefore, subsequent investigations will focus primarily on the ITC of the Top configuration.

To further explore the active regulation mechanism of interfacial thermal transport, compressive strain was applied to modulate the ITC of HEA/Gr/HEA heterojunctions, with results shown in Figs. 2c and d. As shown in Fig. 2c, the heterostructure exhibits a typical elastoplastic mechanical response under compressive loading: when the compressive strain increases from 0 to approximately 4.3%, the stress increases linearly, indicating elastic deformation; thereafter, the stress growth rate slows down, entering the yielding and plastic flow stage, with stress approaching saturation at a strain of about 8.5%. Based on this, the present study focuses on the mechanism by which compressive strain affects ITC within the elastic deformation regime (strain range of 0–4%). The plastic deformation regime (beyond ~ 4.3% strain) lies outside the scope of this work, as irreversible structural changes (e.g., dislocation nucleation, atomic rearrangement, and potential interfacial debonding) would introduce complex microstructural evolution that cannot be captured by the current quasi‑static analysis [48–50]. As shown in Fig. 2d, within the elastic strain range, ITC exhibits a linear increasing trend with compressive strain. Specifically, when the compressive strain increases from 0 to 4%, ITC rises from 185.2 MW/m^2^K to 353.4 MW/m^2^K, an increase of approximately 90.8%. It is proposed that this enhancement primarily originates from the compressive strain‑induced increase in interfacial compressive stress, which tightens the van der Waals contact between the high‑entropy alloy and the graphene layers and strengthens atomic interactions at the interface, thereby improving the interfacial coupling efficiency of heat carriers (mainly phonons) and significantly enhancing interfacial thermal transport capability. Notably, as indicated by the blue comparison curve in Fig. 2d, applying only 2% compressive strain completely compensates for the interfacial thermal transport degradation caused by void defects, bringing the system to the interfacial thermal transport level of a defect‑free HEA/Gr/HEA heterostructure. This result demonstrates that compressive strain effectively strengthens atomic coupling in the interfacial region by modulating the local stress field around the nanopores, thereby achieving recovery and enhancement of ITC. In summary, compressive strain not only serves as an effective means of modulating ITC but also exhibits potential for compensating defect‑induced thermal resistance. It should be noted that the 2% compensation threshold is demonstrated here only for the Top‑9 configuration. Based on the ITC data in Fig. 2b, the degradation severity varies with defect type (Both being the most severe, Top being the least) and pore number. Therefore, the strain required for full compensation likely depends on the specific defect configuration. A systematic investigation of strain compensation across different defect types and pore numbers is warranted in future work.

The temperature dependence of ITC is important for practical device applications [51]. We have now performed additional NEMD simulations for the HEA/Gr/HEA_Top‑9 configuration at elevated temperatures (300–800 K). The results of Table 3 show that ITC gradually increases with temperature. Specifically, when the temperature is raised from 300 K to 800 K, the ITC increases from 185.2 MW/m^2^K to 243.9 MW/m^2^K, an enhancement of 31.7%. This is consistent with previous reports that elevated temperatures promote interfacial phonon thermal transport [41, 52, 53]. Further analysis indicates that at higher temperatures, the phonon population increases significantly, providing more heat carriers available to participate in interfacial transport. More importantly, elevated temperatures activate additional anharmonic phonon scattering channels (e.g., three‑phonon processes), which enhance inelastic phonon transmission across the interface. This increased anharmonic coupling facilitates energy exchange between the HEA and graphene layers even when their phonon spectra are not perfectly matched. In other words, while anharmonic scattering reduces in‑plane phonon mean free paths within each material (thereby decreasing the bulk thermal conductivity), it simultaneously opens additional pathways for heat to cross the interface, leading to an increase in ITC.

**Table 3:** Effect of temperature on the ITC of the HEA/Gr/HEA_Top-9 structure

Temperature (K)	ITC (MW/m^2^K)
300	185.2
400	206.7
500	218.6
600	233.4
700	238.9
800	243.9

### Analysis of the underlying mechanism of thermal regulation

**Fig. 3:**
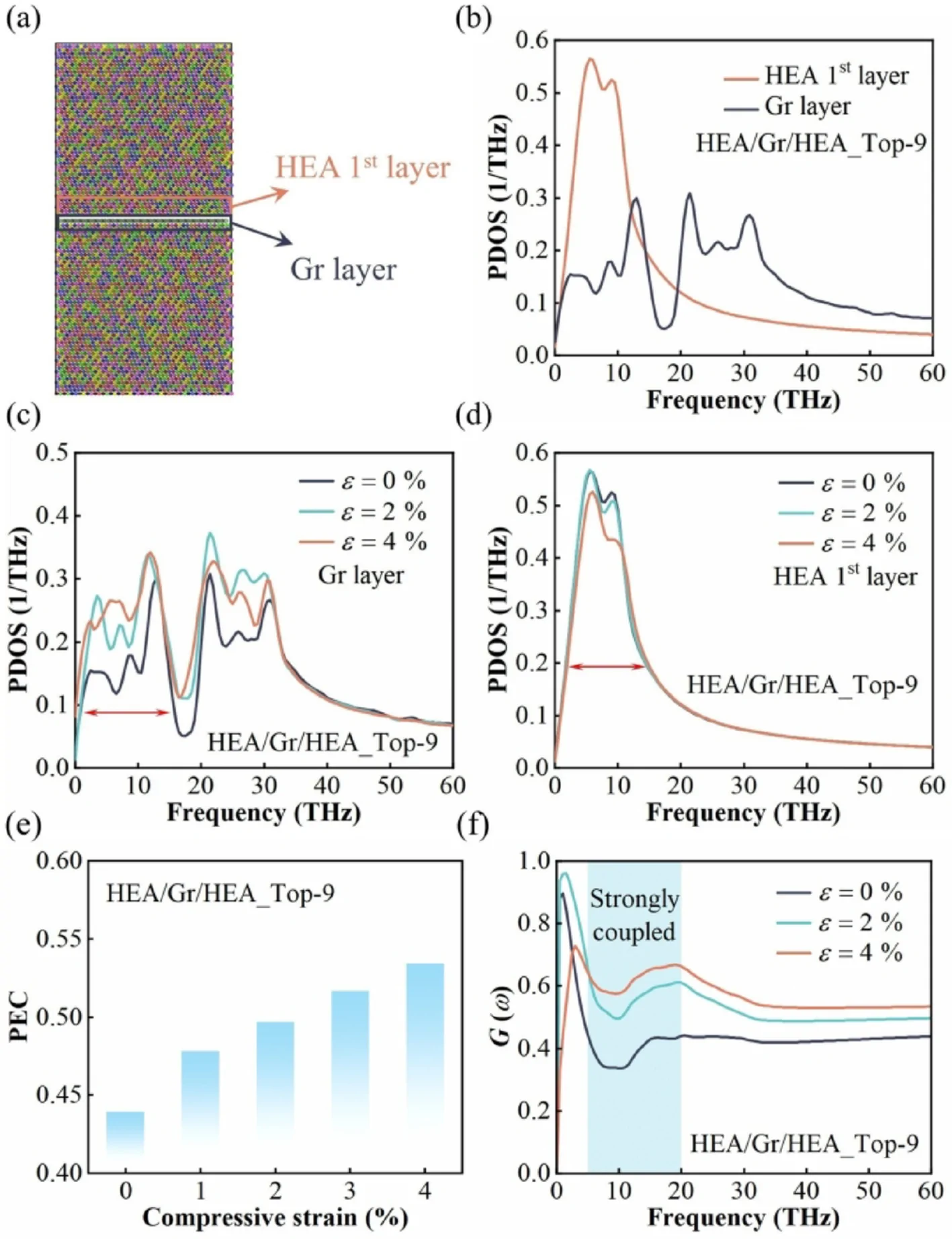
Analysis of the intrinsic phonon mechanism.** a** schematic of the near-interface atomic layer.** b** PDOS distributions of the HEA layer and Gr in the pristine HEA/Gr/HEA_Top-9 configuration.** c** PDOS variation of the Gr layer and** d** PDOS variation of the HEA layer under compressive strain.** e** variation of PEC with increasing compressive strain.** f** variation of PCSD with frequency under different compressive strains

To further elucidate the microscopic physical mechanism by which compressive strain enhances the ITC of HEA/Gr/HEA heterojunctions, a systematic analysis is conducted from the perspective of phonon dynamics, with the calculated results presented in Fig. 3. The HEA/Gr/HEA_Top-9 configuration, which exhibits superior thermal transport performance compared to other interface structures, is selected as the representative model for in-depth investigation. Figure 3b shows the PDOS of the HEA and Gr layers on both sides of the interface under zero compressive strain (*ε* = 0%). Here, the HEA layer is taken as an atomic layer approximately 10 Å from the interface to reflect the vibrational characteristics of the near-interface region; a schematic of this near-interface atomic layer is shown in Fig. 3a. The results show that the PDOS of the HEA layer is mainly concentrated in the low-frequency range of 0–15 THz, with its peak intensity significantly higher than the PDOS of the Gr layer within this frequency band. Specifically, the HEA layer exhibits strong low-frequency phonon activity in the range of 0–10 THz, with a cutoff frequency of approximately 20 THz. In contrast, the Gr layer displays a broader phonon-active frequency band, covering the low-to-medium frequency range of 0–20 THz and the medium-to-high frequency range of 20–40 THz, with a cutoff frequency significantly higher than that of the HEA layer. This difference implies that the Gr layer can excite more high-frequency phonon modes to participate in the thermal transport process. Previous studies have shown that at the micro/nanoscale, the efficiency of interfacial thermal transport mainly depends on the degree of phonon vibrational matching between the materials on both sides of the interface, with the PDOS overlap integral commonly used as a quantitative evaluation metric [54, 55]. Furthermore, low-frequency phonons, owing to their longer wavelengths and higher penetration capability, tend to contribute stronger interfacial phonon coupling. Therefore, in the HEA/Gr/HEA system, the degree of PDOS overlap in the low-frequency region of 0–15 THz is considered the key factor governing interfacial thermal transport. When compressive strain is applied, the PDOS distribution changes significantly, as shown in Fig. 3c (*ε* = 2%) and Fig. 3d (*ε* = 4%). As the compressive strain increases from 0% to 4%, the PDOS of the Gr layer in the low-frequency region of 0–15 THz exhibits a gradual upward trend. In contrast, the PDOS of the HEA layer within the same frequency band gradually weakens. This opposite evolution behavior continuously improves the PDOS overlap between HEA and Gr in the low-frequency region, thereby gradually enhancing interfacial phonon coupling. To quantitatively evaluate the change in phonon coupling strength, the PEC and PCSD of the HEA/Gr/HEA_Top-9 configuration are calculated, with results shown in Figs. 3e and f. The analysis indicates that under compressive strain, the phonon energy coupling across the interface is significantly enhanced. Specifically, when the compressive strain increases from 0% to 4%, the PEC value increases from 0.44 to 0.53, a relative increase of 20.45%. Further combining the frequency-domain distribution of PCSD reveals that the increase in PEC mainly originates from the enhancement of phonon coupling intensity in the 5–20 THz frequency band. This frequency band covers part of the low-frequency vibrational modes of HEA and the medium-frequency vibrational modes of Gr; the synergistic enhancement of these modes effectively promotes interfacial thermal transport capability. Therefore, compressive strain, by modulating the PDOS distributions of HEA and Gr in the low-to-medium frequency regions, increases their phonon spectral overlap, thereby strengthening interfacial phonon coupling and ultimately enhancing the interfacial thermal transport capacity of the HEA/Gr/HEA heterojunction.

While the PDOS and PEC analyses reveal enhanced phonon spectral overlap under compressive strain, the underlying atomic‑scale structural changes remain to be fully elucidated. We note that compressive strain likely induces out‑of‑plane rippling or buckling in the graphene layer, which can soften the flexural (ZA) phonon modes and increase their low‑frequency spectral weight [56, 57]. Simultaneously, compression densifies the high‑entropy alloy near the interface, leading to stiffening of its vibrational modes [58, 59]. However, a quantitative correlation of these structural changes with the observed PDOS evolution would require detailed atomic displacement analysis (e.g., mode‑projected phonon spectra and real‑space ripple amplitude measurements), which is beyond the scope of the present work. Therefore, our current conclusions remain phenomenological regarding the direct structural origin of the enhanced phonon coupling.

**Fig. 4:**
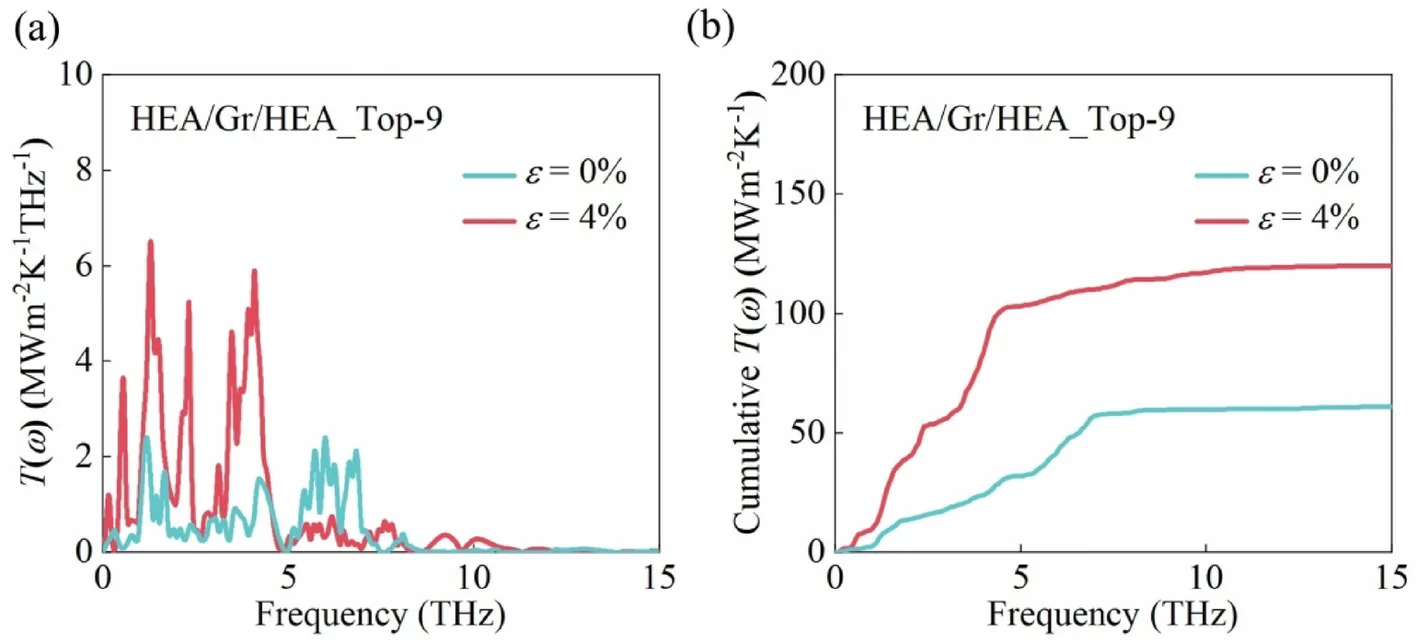
Spectral heat flux (SHC) analysis, with frequency-resolved spectral ITC and cumulative spectral ITC varying with frequency.** a** the influence of 4% strain on SHC.** b** the variation of cumulative spectral interfacial thermal conductance under 4% strain modification

In addition, we further numerically calculated the SHC of the HEA/Gr/HEA heterojunction and analyzed the effect of interfacial phonon transport on ITC in the frequency domain, as shown in Fig. 4. The results indicate that the high-density distribution of low-frequency phonons in the 0–10 THz range provides the main channel for interfacial heat transfer. This is consistent with the strong coupling phenomenon of PDOS in the 0–10 THz low-frequency range. For the pristine interface (HEA/Gr/HEA_Top-9), the spectral heat flux confirms that low-frequency acoustic phonons (below 20 THz) carry the majority of the energy, a common characteristic of van der Waals interfaces. The introduction of 4% strain induces a remarkable enhancement within the 0–15 THz range. This enhancement aligns perfectly with the increased overlap in the PDOS we observed, unequivocally demonstrating that 4% strain boosts ITC by facilitating more efficient coupling and transmission of these low-frequency modes. The spectral heat flux analysis, therefore, provides the definitive microscopic picture that reconciles the impacts of strain on interfacial thermal transport.

**Fig. 5:**
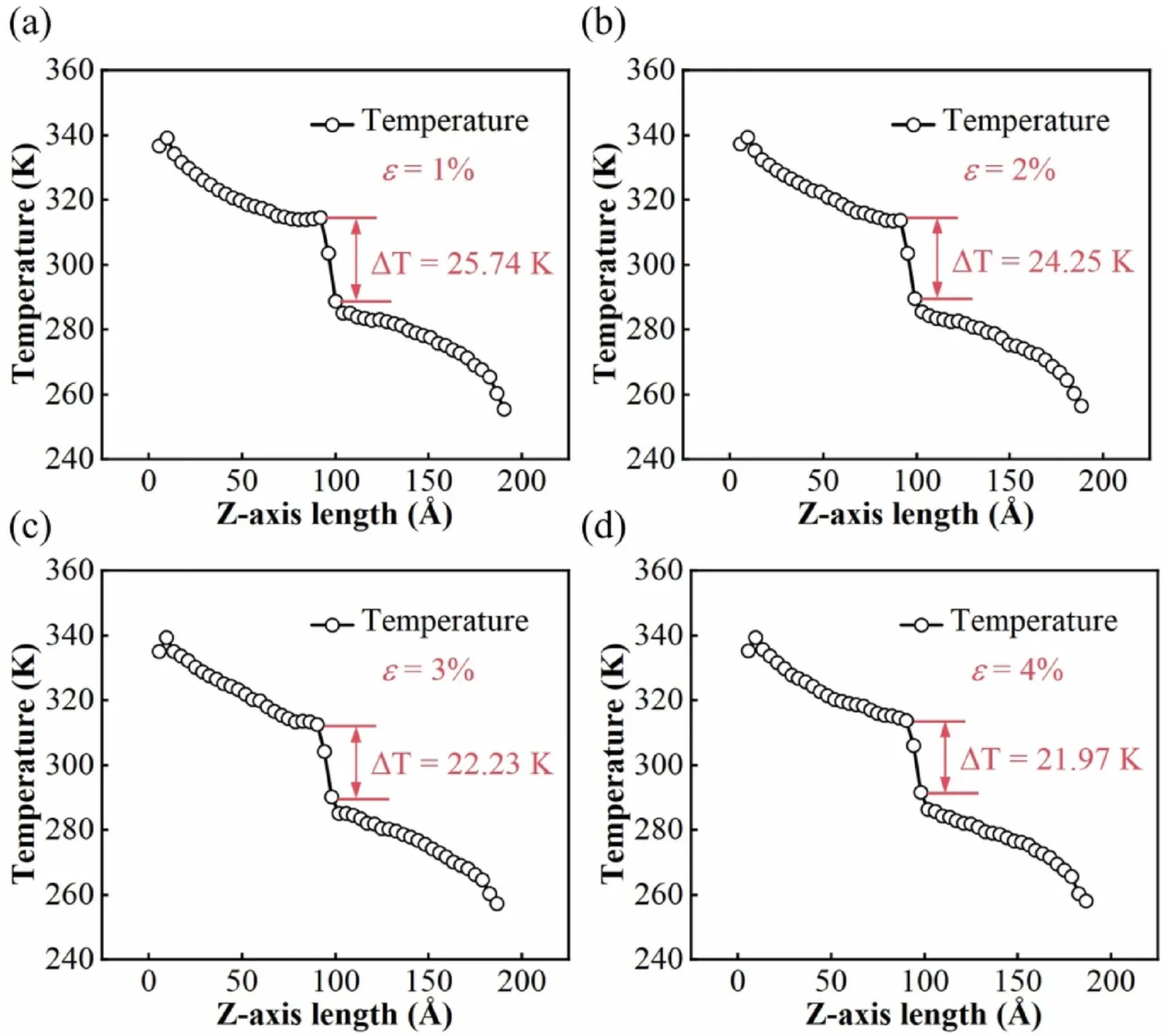
Temperature gradient distributions of the HEA/Gr/HEA_Top-9 heterojunction under compressive strain.** a** 1%.** b** 2%.** c** 3%.** d** 4%

The magnitude of ITC depends to a large extent on the amplitude of the temperature jump (ΔT) at the interface, a classical theoretical understanding in micro/nanoscale thermal transport research [60, 61]. Based on this theoretical framework, the temperature jump behavior of the interfacial layer under different compressive strain conditions is systematically analyzed for the HEA/Gr/HEA_Top-9 heterojunction with a void-defective interface, aiming to reveal the intrinsic mechanism of ITC variation under compressive strain modulation from the perspective of actual heat flux transport; the results are shown in Fig. 5.

To explicitly characterize the interface region, we define the interfacial width as the distance over which the temperature profile deviates from the linear bulk trends. Under steady-state conditions, this width is approximately 5 Å on each side of the Gr/HEA interface. Notably, the definition of interfacial width does not affect the measured steady-state temperature gradient, as the gradient is determined by the bulk thermal conductivity and the imposed heat flux, not by the atomic-scale interface width [62–64]. Instead, the key interfacial quantity governing thermal transport is the temperature jump (ΔT) across the interface. We have verified that varying the interfacial width by ± 5 Å alters ΔT by less than 3%, confirming the robustness of our results. With this clarification, we now analyze the temperature jump behavior under different compressive strains. Figures 5a and 4d show the temperature gradient distributions along the heat flux direction of the HEA/Gr/HEA_Top-9 heterojunction at compressive strains of 1%, 2%, 3%, and 4%, respectively. It can be observed that as the compressive strain increases, the amplitude of the temperature jump across the interface exhibits a gradually decreasing trend. Specifically, at 1% strain (Fig. 5a), there is a clear temperature discontinuity at the interface, with a temperature jump value of approximately 25.74 K, indicating high interfacial thermal resistance and significant hindrance to heat transfer across the interface. When the compressive strain increases to 2% and 3% (Figs. 5b and 4c), the temperature jump amplitude gradually narrows and decreases to approximately 22.23 K, and the temperature profiles on both sides of the interface become more continuous, reflecting an improvement in interfacial thermal transport efficiency. When the strain is further increased to 4% (Fig. 5d), the interfacial temperature jump reaches its lowest value, approximately 21.97 K, and the variation of the temperature gradient at the interface becomes the smoothest. This monotonic decreasing trend of the temperature jump with increasing compressive strain (from 25.74 K to 21.97 K, a reduction of approximately 17.16%) is highly consistent with the aforementioned increasing trend of ITC with strain (PEC increasing from 0.44 to 0.53, an increase of 20.45%). This consistency strongly indicates that in the HEA/Gr/HEA_Top-9 heterojunction, compressive strain enhances ITC primarily by reducing the interfacial temperature jump. The physical origin is that compressive strain increases the spectral overlap of low- to medium-frequency phonons between the HEA and Gr layers on both sides of the interface, promotes phonon energy coupling, and enables more heat flux to cross the interface with lower scattering loss, thereby reducing the temperature jump amplitude and ultimately achieving a significant improvement in ITC. In summary, the quantitative analysis of the interfacial temperature jump further validates the microscopic mechanism by which compressive strain optimizes the interfacial thermal transport capacity of HEA/Gr/HEA heterojunctions. Compressive strain induces an increase in phonon spectral overlap, which in turn enhances interfacial phonon coupling, reduces the interfacial temperature jump, and drives the improvement of ITC.

## Conclusions

This paper systematically investigates the interfacial thermal transport performance of HEA/Gr/HEA heterojunctions containing void defects and the regulatory mechanism of compressive strain using non-equilibrium molecular dynamics simulations. The main conclusions are as follows:

1. Void defects significantly suppress thermal transport. In the defect-free case, the through-plane thermal conductivity is approximately 1.25 W/mK, and the ITC is approximately 212.3 MW/m^2^K. When the number of nanopores increases to nine, the ITC decreases to approximately 139.6 MW/m^2^K (a reduction of 34.2%), with the configuration containing nanopores on both sides of the interface exhibiting the most severe degradation, attributed to the enhanced phonon scattering effect induced by the nanopores.
2. Compressive strain can effectively enhance and restore ITC. Within the elastic strain range of 0–4%, ITC increases linearly from 185.2 to 353.4 MW/m^2^K, an increase of approximately 90.8%. Applying only 2% strain completely compensates for the thermal conductivity degradation caused by void defects.
3. The enhancement of interfacial phonon coupling by compressive strain is the core mechanism. The strain increases the PDOS of the Gr layer in the low-frequency region of 0–15 THz while decreasing that of the HEA layer, thereby improving their spectral overlap. The phonon energy coupling coefficient increases from 0.44 to 0.53 (an increase of 20.45%).
4. Compressive strain improves interfacial thermal transport performance by reducing the interfacial temperature jump. When the strain increases from 1% to 4%, the interfacial temperature jump decreases from 25.74 K to 21.97 K (a reduction of 17.16%), consistent with the increasing trend of ITC.

The findings of this work provide a theoretical basis for the thermal management of high-entropy alloy/graphene heterojunctions and for strain-modulated interfacial thermal resistance.

## Data Availability

All data supporting the findings of this study are available within the paper and its Supplementary Information.
